# The association between long-term outdoor air pollution exposure and Chinese visceral adiposity index: A nationwide study of middle-aged and older adults

**DOI:** 10.1371/journal.pone.0325524

**Published:** 2025-07-17

**Authors:** Dong Liu, Xiaoyan Luo, Kunyan Zhou

**Affiliations:** 1 Department of Obstetrics and Gynecology, West China Second University Hospital, Sichuan University, Chengdu, China; 2 Key Laboratory of Birth Defects and Related Diseases of Women and Children (Sichuan University), Ministry of Education, China; 3 Hi-Tech Zone Hospital for Women and Children, West China Second University Hospital, Sichuan University, China; Freelance Medical Research and Writing, UNITED KINGDOM OF GREAT BRITAIN AND NORTHERN IRELAND

## Abstract

Outdoor air pollutants, particularly particulate matter (PM) and nitrogen dioxide (NO_2_), have been closely linked to diabetes mellitus, other metabolic disorders, and cardiovascular diseases. Visceral adiposity, a common high-risk factor for these conditions, may mediate the impact of air pollution on disease development. However, the potential role of outdoor air pollution on visceral adiposity remains unclear, especially in the Asian population and in older adults (> 60 years). Given the high levels of air pollution and the rising prevalence of visceral obesity in China, this study investigated whether there is a linear and/or non-linear association between the Chinese Visceral Adiposity Index (cVAI) and exposure to individual and combined air pollutants in 7,552 participants aged ≥ 45 years in China. Data on air pollutants were acquired from the ChinaHighAirPollution dataset. These included PM with an aerodynamic diameter ≤ 2.5 μm (PM_2.5_), PM_10_, NO_2_, ozone (O_3_), and sulfur dioxide (SO_2_). Our results demonstrate that exposure to all of these pollutants (PM_2.5_, PM_10_, NO_2_, O_3_, and SO_2_) was significantly and positively associated with the cVAI, with dose-response relationships observed in trends test across pollutants. In subgroup anslysis, the associations were particularly pronounced in men and active smokers. Specifically, smokers in the highest quartile of PM_2.5_ exposure had a β coefficient of 16.89 (95%CI:11.00, 22.78), while males had a β coefficient of 14.38 (95%CI:9.68, 19.07). NO_2_ and PM_2.5_ were identified as the primary contributors to the total effect of outdoor air pollution exposure. In conclusion, this study is the first to reveal that outdoor air pollutants, particularly PM_2.5_ and NO_2_, are significantly associated with increased visceral adiposity in middle-aged and older Chinese adults. Males and active smokers could be high risk groups, when compared with females and non-smokers. This study highlights an urgent need for public health policies mitigating visceral obesity through the reduction of outdoor air pollution exposure.

## Introduction

Obesity, particularly visceral obesity, is strongly associated with the development of various non-communicable diseases, including type 2 diabetes, metabolic syndrome, and cardiovascular diseases [1]. As the global prevalence of obesity continues to rise, this condition has become a significant public health challenge, especially for middle-aged to older adult populations [2]. Although the body mass index (BMI) is widely used as a traditional measure of obesity, this index fails to provide an accurate reflection of fat distribution, especially visceral fat [3]. Visceral fat is a metabolically active tissue that secretes pro-inflammatory cytokines and hormones, and visceral fat accumulation is more strongly associated with metabolic and cardiovascular disorders than BMI [4]. Over the recent years, the visceral adiposity index (VAI) has been widely employed to evaluate the impact of visceral fat on metabolic diseases. However, the VAI was poorly related to the adipose tissue area in individuals of Chinese origin [5], as the VAI was originally developed in a Caucasian population, and its applicability to Chinese populations remains controversial due to significant differences in body fat distribution and metabolic profiles between these ethnic groups [6,7]. Consequently, the Chinese VAI (cVAI) was developed to evaluate the distribution of visceral fat in populations of Chinese ancestry specifically [8]. Indeed, compared with other obesity indices, the cVAI demonstrated the best predictive performance for diabetic complications, cardiovascular disease (CVD), prediabetes and diabetes in Chinese people, notably the metabolically unhealthy normal weight and metabolically healthy obese individuals of the China Health and Retirement Longitudinal Study (CHARLS) [9,10].

A significant environmental contributor to the global burden of cardiometabolic disease is outdoor air pollution, particularly in low- and middle-income countries (LMICs) where pollution levels continue to escalate. Accordingly, outdoor air pollution exposure has been associated with many proxies of cardiometabolic disease. However, only a small number of published studies have investigated the association between outdoor air pollution exposure and visceral adiposity specifically in LMICs such as China [11,12]; and to the best of our knowledge, visceral adiposity was not measured by the cVAI in any of those studies.

The increasing proportion of middle-aged to older adults in China [13] and the heavier burden of outdoor air pollution and cardiometabolic diseases in middle-aged to older adults than in younger adults [14] highlight a contemporary need for studies assessing whether there is an association between outdoor air pollution exposure and the cVAI. We therefore conducted a study to investigate the association between multiple air pollutants (PM_2.5_, PM_10_, NO_2_, O_3_, and SO_2_) and the cVAI in middle-aged and older adults in China. This study provides evidence to support future environmental policies such as the scientific establishment of safety thresholds for the different air pollutants assessed and the adoption of more energy-efficient and eco-friendly industrial and lifestyle measures to reduce the pollutant concentrations in the environment and thus ultimately benefit the public health sector.

## Methods

### Study design, data source, and study population

This study report is based on the STrengthening the Reporting of OBservational studies in Epidemiology (STROBE) statement for the cross-sectional study design. The data analyzed in this cross-sectional study were acquired from CHARLS, a nationally representative prospective cohort study that was originally designed to evaluate the health and socioeconomic status of adults aged 45 years and above in China using face-to-face computer-assisted personal interviews [15]. CHARLS includes the following data for each participant: demographics, family structure, health status, health care and insurance, biomarkers, work, retirement, income and consumption, health-related behaviors, and residence. Those data are mentioned in the CHARLS handbook available at http://charls.pku.edu.cn/en. Detailed descriptions of the CHARLS methods have been published previously [16]. The CHARLS study uses stratified sampling and probability-proportional-to-size (PPS) sampling techniques and covers 150 district-level units and 450 village-level units within communities or villages across 126 cities and counties in 28 provinces of China. Only participants with an age ≥ 45 years on the identification card and willing to participate in the survey were included in CHARLS. The CHARLS dataset is highly representative of the population and provides an accurate reflection of the overall conditions of the older adult population in both urban and rural areas across China [15]. In addition, the CHARLS study is conducted in accordance with the tenets of the Declaration of Helsinki and was approved by the Peking University’s Ethics Review Committee (Reference: IRB00001052–11015).

The first wave of initial patient surveys was in 2011, followed by subsequent waves every two to three years. To date, five waves of participant enrollment have been completed, encompassing 2011, 2013, 2015, 2018, and 2020. As data from the 2018 and 2020 waves lacked the anthropometric and metabolic parameters needed to calculate the cVAI, this study is based on data from the 2015 wave which is the most recent CHARLS dataset allowing the assessement of the cVAI. The exclusion criteria were (S1 Fig): (1) Actual age < 45 years, (*n* = 1932 participants); (2) missingness of information on outdoor air pollution exposure (*n* = 5543 participants); (3) missingness of information about the parameters necessary to calculate the cVAI (height, weight, waist circumference, and serum concentrations of triglycerides and high density lipoprotein cholesterol (HDLc) (*n* = 4997 participants) and (4) missingness of information on covariates in the assessment of the association between outdoor air pollution exposure and cVAI, including education, active smoking, and alcohol consumption (*n* = 1071 participants).

### Measurement of long-term outdoor air pollution exposure

We assessed five air pollutants known to harm human health, increasing the susceptibility to cardiometabolic disorders and cancers among others: particulate matter (PM) with an aerodynamic diameter ≤ 2.5 μm (PM_2.5_), PM with an aerodynamic diameter ≤ 10 μm (PM_10_), nitrogen dioxide (NO_2_), ozone (O_3_), and sulfur dioxide (SO_2_) [17–19]. Ambient PM_2.5_, PM_10_, NO_2_, O_3_ and SO_2_ mean concentrations in 2015 were obtained from the ChinaHighAirPollution (CHAP) dataset of the National System Science Data Center (https://www.geodata.cn), a high-resolution dataset integrating satellite and ground-based observations with model data. To increase the precision of our estimates on outdoor air pollution exposure, we considered a spatial resolution of 10x10 km for SO_2_, and 1x1 km for the other pollutants assessed including PM_2.5_, PM_10_, and O_3_. The 10-fold cross-validation model demonstrated high accuracy and predictive power, suggesting that the estimated levels of air pollutants in this study aligned closely with ground-based measurements, as demonstrated by coefficients of determination ranging from 0.8 to 0.92. For each participant, outdoor air pollution exposure was assessed by geocoding their residential address and assigning the corresponding pollutant concentrations.

### cVAI measurement

Sex-specific cVAIs were calculated using the formulas provided below:

Male cVAI = -267.93 + 0.68 × age (years) + 0.03 × BMI (kg/m^2^) + 4.00 × waist circumference (cm) + 22.00 × log [triglycerides (mmol/L)] - 16.32 × HDL (mmol/L)

Female cVAI = −187.32 + 1.71 × age (years) + 4.23 × BMI (kg/m^2^) + 1.12 × waist circumference (cm) + 39.76 × log [triglycerides (mmol/L)] – 11.66 × HDL (mmol/L). Height, weight and waist circumference measurements were collected by two trained surveyors. Venous blood samples were drawn from participants after an overnight fasting period, stored in anticoagulant tubes, and then sent to the laboratory for standard assessment of HDLC, triglycerides, glycated hemoglobin (HbA1C), low density lipoprotein cholesterol (LDLc), glucose, blood urea nitrogen (BUN), creatinine, uric acid and cystatin C levels. For detailed information on data collection and the laboratory methods of testing, please refer to the CHARLS Handbook available at: https://charls.pku.edu.cn/.

### Statistical analysis

Statistical analyses were performed using the R software (Version 4.4.1; R Foundation for Statistical Computing, Vienna, Austria, https://www.r-project.org/). A multivariable linear regression analysis was applied to evaluate the associations between the concentrations of the individual air pollutants and the cVAI. The outdoor air pollutant concentrations considered as the independent variables were categorized into quartiles, whereas the cVAI was the dependent variable. Three multivariable linear regression models were constructed: (1) a Crude Model (unadjusted); (2) Model 1 adjusted for sociodemographic factors (age, sex, locality of residence, region of residence and self-reported level of education and marital status, and (3) Model 2 further adjusted for lifestyle factors (self-reported smoking status and alcohol consumption), self-reported health status, hypertension, and physical disability. The covariates assessed have previously been correlated with both outdoor air pollution exposure and the cVAI in real-world scenarios [20,21]. The sex was a binary variable (women and men), corresponding to the sex assigned at birth. The self-reported marital status was divided into three categories: married, divorced and never been married. Self-reported alcohol consumption was categorized as no, once per month and more than once per month. Because every individual admitted to the Education system in China must complete at least 9 years of school, the self-reported level of education was categorized as illiterate, middle school or below and high school or above. The diagnosis of hypertension was based on participants’ self-reports or on the persistence of systolic blood pressure values ≥140 mmHg and/or diastolic blood pressure values ≥ 90 mmHg recorded three times on the same day. Besides the sex, the age (45 years-60 years and > 60 years), locality of residence (urban and rural areas), region of residence (divided by the Qinling mountain into the North region combining a cold and dry climate with a heavy industrial and traditional manufacturing-based economy, and the South region combining a warm and humid climate with a high-tech industrial and light manufacturing-based economy) and self-reported smoking status (yes and no), health status (good and poor) and physical disability (yes and no) were binary variables. Trends tests were conducted to investigate whether there were dose response relationships across quartiles. To assess the robustness of our findings, we conducted a two-stage sensitivity analysis. The first stage consisted of adjusting for the BMI in addition to the other covariates, and the second stage consisted of data imputation for the missing covariates. Missing categorical covariates were coded as “absent”, whereas median values were used for the imputation of continuous covariates. Subgroup analyses were subsequently performed to evaluate the influence of each of the above-mentioned covariates on the linear relationship between air pollutant concentrations and cVAI. A covariate was deemed to be a statistically significant influencing factor of the relationship between an air pollutant level and cVAI when the p-value for the test of the impact of that covariate on the relationship was less than 0.05. In addition, restricted cubic spline regression models featuring four nodes (5th, 35th, 65th, and 95th percentiles) were used to investigate whether the association between the pollutant concentrations and cVAI was also non-linear. Moreover, we applied the weighted quantile sum (WQS) regression analysis [22] to evaluate the relative contribution of each pollutant to the overall combined effect of outdoor air pollution given the simultaneous exposure to multiple air pollutants in real-world scenarios.

The participants’ characteristics were compared across sexes using parametric or non-parametric tests for continuous variables and Chi-squared or Fischer exact tests for categorical variables, with p-values less than 0.05 indicating potentially statistically significant differences. Continuous variables are summarized as means ± standard deviation (SD) while categorical variables are reported as percentages. The result presentation is enhanced by Figures and tables.

## Results

### Characteristics of the study population

In this study, we analyzed 7,552 participants (including 3,463 men and 4,089 women of mean age 61.3 years old; S1 Fig). 51.6% of the participants were aged over 60 years, while 48.4% were aged between 45 and 60 years old. Women had a higher mean BMI (24.5 ± 3.9 vs. 23.6 ± 3.5 kg/m^2^) and cVAI (108.2 ± 34.7 vs. 102.8 ± 48.2) values than men (*p* < 0.0001). The frequency of active smoking was significantly higher in men (52.8%) than in women (6.2%, *p* < 0.0001). The majority of participants were married (87.3%), resided in rural areas (61.8%), and had a self-reported poor health (75.9%). The characteristics of participants are summarized in Table 1.

**Table 1:** Summary characteristics of study participants stratified by sex.

Variable	All participants (*n* = 7552)	Men (*n* = 3463)	Women (*n* = 4089)	*p*-value
Age (years)	61.3 ± 9.1	62.1 ± 9.2	60.6 ± 9.1	< 0.0001
Age group, n (%)				< 0.0001
> 60	3898 (51.6)	1919 (55.4)	1979 (48.4)	
≤ 60	3654 (48.4)	1544 (44.6)	2110 (51.6)	
BMI (kg/m^2^)	24.1 ± 3.8	23.6 ± 3.5	24.5 ± 3.9	< 0.0001
cVAI	105.7 ± 41.5	102.8 ± 48.2	108.2 ± 34.7	< 0.0001
Self-reported marital status, n (%)				< 0.0001
Never married	37 (0.5)	35 (1.0)	2 (0.1)	
Married	6597 (87.4)	3119 (90.1)	3478 (85.1)	
Divorced	918 (12.2)	309 (8.9)	609 (14.9)	
Self-reported level of education, n (%)				< 0.0001
Illiterate	1840 (24.4)	369 (10.7)	1471 (36.0)	
Middle school or below	4817 (63.8)	2529 (73.1)	2288 (56.0)	
High school or above	895 (11.9)	565 (16.3)	330 (8.1)	
Region of residence, n (%)				0.75
North	4254 (56.3)	1958 (56.5)	2296 (56.2)	
South	3298 (43.7)	1505 (43.5)	1793 (43.9)	
Locality of residence, n (%)				0.04
Rural area	4669 (61.8)	2185 (63.1)	2484 (60.8)	
Urban area	2883 (38.2)	1278 (36.9)	1605 (39.3)	
Self-reported alcohol consumption, n (%)				< 0.0001
No	4924 (65.2)	1442 (41.6)	3482 (85.2)	
> 1 m	1939 (25.7)	1623 (46.9)	316 (7.7)	
< 1 m	689 (9.1)	398 (11.5)	291 (7.1)	
Self-reported smoking status, n (%)				< 0.0001
No	5469 (72.4)	1635 (47.2)	3834 (93.8)	
Yes	2083 (27.6)	1828 (52.8)	255 (6.2)	
Self-reported health status, n (%)				< 0.0001
Poor	5732 (75.9)	2554 (73.8)	3178 (77.7)	
Good	1820 (24.1)	909 (26.3)	911 (22.3)	
Hypertension, n (%)				0.25
No	4166 (55.2)	1885 (54.4)	2281 (55.8)	
Yes	3386 (44.8)	1578 (45.6)	1808 (44.2)	
Self-reported physical disability, n (%)				< 0.0001
No	7342 (97.2)	3331 (96.2)	4011 (98.1)	
Yes	210 (2.8)	132 (3.8)	78 (1.9)	

### Characteristics of long-term outdoor air pollution exposure

**Fig 1:**
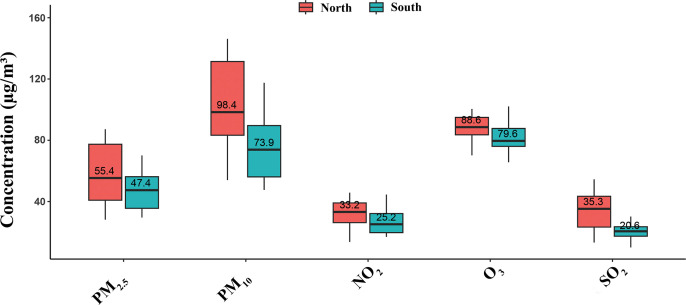
Geographic distribution of the air pollutant concentrations in cities in the North and South regions of China.

The mean annual concentrations of PM_2.5_, PM_10_, NO_2_, O_3_, and SO_2_ were 52.8 µg/m^3^, 89.3 µg/m^3^, 29.7 µg/m^3^, 85.8 µg/m^3^, and 28.1 µg/m^3^, respectively (Table 2). All these concentrations exceeded the World Health Organization (WHO) safety thresholds, and PM_2.5_ and PM_10_ further exceeded the safety thresholds (35 µg/m^3^ and 70 µg/m^3^, respectively) in China specifically [23,24]. Notably, the levels of pollutants were consistently higher in Northern than Southern cities: PM_2.5_ (55.4 vs. 47.4 μg/m³), PM_10_ (98.4 vs. 73.9 μg/m³), NO_2_ (33.2 vs. 25.2 μg/m³), O_3_ (88.6 vs. 79.6 μg/m³), and SO_2_ (35.3 vs. 20.6 μg/m³) (Fig 1).

**Table 2:** Summary statistics of the estimated concentrations of the air pollutants PM_2.5_, PM_10_, NO_2_, O_3_ and SO_2_.

Pollutant (μg/m^3^)	Mean	GM	5th	25th	Median	75th	95th	IQR
PM_2.5_	52.8	50.3	30.0	39.6	51.5	62.3	83.1	22.7
PM_10_	89.3	84.8	50.3	63.7	88.0	107.7	140.1	44.0
NO_2_	29.7	28.4	16.2	21.0	29.4	36.7	45.3	15.6
O_3_	85.8	85.4	72.6	78.6	84.9	93.5	98.9	14.9
SO_2_	28.1	25.8	13.7	19.5	23.7	39.6	50.0	20.2

Abbreviations: GM, geometric mean; IQR, interquartile range.

### Association between individual air pollutant concentrations and cVAI

#### Multivariable linear regression analysis.

Results of the mulivariable linear regression analysis of the association between air pollutant concentrations and cVAI are presented in Table 3. In model 2, higher PM_2.5_, PM_10_, NO_2_, SO_2_, and O_3_ concentrations were significantly associated with an increased cVAI (all *p* for trend < 0.0001). We found a linear relationship between cVAI and PM_2.5_, PM_10_, NO_2_, O_3_ and SO_2_ concentrations, as shown in Table 3 by β coefficients increasing from Q1 to Q4 concentrations for all the pollutants (*p* < 0.0001).

**Table 3:** Results of the multivariable regression analysis of the association between air pollution exposure based on quartiles of pollutants’ concentrations and cVAI.

			cVAI			
	Crude model		Model 1		Model 2	
	β (95%CI)	*p*	β (95%CI)	*p*	β (95%CI)	*p*
**PM**_**2.5**_						
Q1	ref		ref		ref	
Q2	6.1 (3.4, 8.7)	< 0.0001	5.3 (2.8, 7.9)	< 0.0001	5.5 (3.1, 8.0)	< 0.0001
Q3	9.7 (7.1, 12.3)	< 0.0001	9.6 (7.1,12.2)	< 0.0001	9.7 (7.3, 12.2)	< 0.0001
Q4	17.0 (14.4, 19.7)	< 0.0001	12.3 (9.5,15.1)	< 0.0001	11.6 (8.9, 14.3)	< 0.0001
*p* for trend		< 0.0001		< 0.0001		< 0.0001
**PM**_**10**_						
Q1	ref		ref		ref	
Q2	4.8 (2.0, 7.3)	< 0.001	3.8 (1.2, 6.3)	0.004	3.58 (1.1, 6.1)	0.005
Q3	10.9 (8.2, 13.4)	< 0.0001	9.2 (6.6, 11.7)	< 0.0001	8.97 (6.5, 11.4)	< 0.0001
Q4	16.6 (13.9, 19.2)	< 0.0001	12.0 (9.1, 14.9)	< 0.0001	11.3 (8.5, 14.01)	< 0.0001
*p* for trend		< 0.0001		< 0.0001		< 0.0001
**NO**_**2**_						
Q1	ref		ref		ref	
Q2	4.36 (1.8, 7.0)	< 0.001	3.7 (1.2, 6.2)	0.004	3.7 (1.2, 6.1)	0.003
Q3	15.0 (12.3, 17.6)	< 0.0001	12.2 (9.6, 14.7)	< 0.0001	11.9 (9.5, 14.4)	< 0.0001
Q4	16.6 (14.0, 19.2)	< 0.0001	12.2 (9.5, 14.9)	< 0.0001	11.2 (8.5, 13.8)	< 0.0001
*p* for trend		< 0.0001		< 0.0001		< 0.0001
**O**_**3**_						
Q1	ref		ref		ref	
Q2	3.1 (0.5, 5.7)	0.02	−0.0 (−2.6, 2.6)	0.97	0.0 (−2.5, 2.6)	0.98
Q3	11.1 (8.4, 13.8)	< 0.0001	6.1 (3.3, 8.8)	< 0.0001	6.1 (3.4, 8.8)	< 0.0001
Q4	13.5 (10.9, 16.1)	< 0.0001	8.1 (5.4, 10.7)	< 0.0001	7.3 (4.7, 9.9)	< 0.0001
*p* for trend		< 0.0001		< 0.0001		< 0.0001
**SO**_**2**_						
Q1	ref		ref		ref	
Q2	6.1 (3.4, 8.7)	< 0.0001	5.8 (3.3, 8.4)	< 0.0001	6.2 (3.7, 8.7)	< 0.0001
Q3	9.8 (7.2, 12.4)	< 0.0001	7.4 (4.8, 10.0)	< 0.0001	7.8 (5.3, 10.4)	< 0.0001
Q4	14.3 (11.6, 16.9)	< 0.0001	11.7 (8.6, 14.7)	< 0.0001	11.0 (8.0, 13.9)	< 0.0001
*p* for trend		< 0.0001		< 0.0001		< 0.0001

Abbreviations: CI, confidence interval; Ref, reference.

Sensitivity analyses confirmed the robustness of our findings, with consistent results observed across all models (Table S1). In stage one sensitive analysis, BMI was adjusted, the highest quartiles of PM_2.5_, PM_10_, NO_2_, O_3_, SO_2_ corresponded to increased cVAI values of 3.8, 2.6, 3.9, 3.1, 3.0 respectively (*p* < 0.001). In stage two sensitive analysis, we reran the analysis with imputed data for missing covariables. The highest quartile of PM_2.5_, PM_10_, NO_2_, O_3_, SO_2_ corresponded to increased cVAI values of 12.1, 11.3, 12.0, 6.7, 10.9 respectively (*p* < 0.0001).

**Fig 2:**
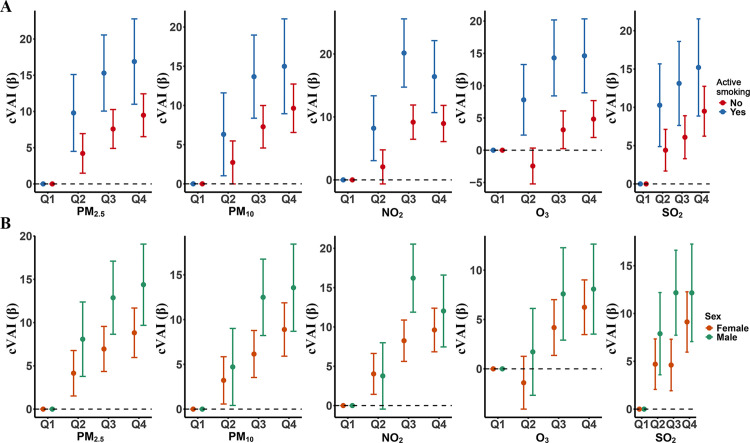
Results of the subgroup analysis of the linear association between pollutant concentrations and cVAI. **A.** Effect of the smoking status. The model was adjusted for covariates other than the smoking status; **B.** Effect of the sex. The model was adjusted for covariates other than the sex.

The factors that significantly influenced the linear relationship between air pollutant concentrations and cVAI were the sex, the smoking status and the region of residence (Table 4). Notably, the magnitude of the association between cVAI and air pollutant concentrations evolved in parallel with quartiles of the pollutant concentrations for PM_2.5_ and PM_10_ in both men and women (Fig 2), but the magnitude of the association was significantly higher in men than women for those two pollutants and NO_2_ (Table 4). Likewise, the magnitude of the association between cVAI and the concentrations of four pollutants (PM_2.5_, PM_10_, NO_2_, and O_3_) was significantly higher in active smokers than in non-smokers (Table 4), with the magnitude of the association evolving in parallel with quartiles of pollutant concentrations for PM_2.5_ and PM_10_ (Fig 2). Moreover, the magnitude of the association between cVAI and the pollutant concentrations was significantly higher in participants residing in the South of China compared with those residing in the North of China for four pollutants (PM_2.5_, PM_10_, SO_2_ and NO_2_), the greatest magnitude being observed for NO_2_.

**Table 4:** Subgroup analyses of the association of air pollutant concentrations with cVAI.

Categories	PM_2.5_			PM_10_			NO_2_			O_3_			SO_2_		
Character	B (95%CI)	*p*	*p* for interaction	β (95%CI)	*p*	*p* for interaction	β (95%CI)	*p*	*p* for interaction	β (95%CI)	*p*	*p* for interaction	β (95%CI)	*p*	*p* for interaction
Age group			0.66			0.9			0.56			0.38			0.74
≤ 60	0.3 (0.2, 0.3)	< 0.0001		0.2 (0.1, 0.2)	< 0.0001		0.5 (0.4, 0.6)	< 0.0001		0.4 (0.2, 0.5)	< 0.0001		0.3 (0.2, 0.5)	< 0.0001	
> 60	0.2 (0.2, 0.3)	< 0.0001		0.2 (0.1, 0.2)	< 0.0001		0.5 (0.4, 0.7)	< 0.0001		0.4 (0.3, 0.6)	< 0.0001		0.3 (0.1, 0.4)	< 0.001	
Sex			0.01			0.003			0.01			0.06			0.05
Male	0.3 (0.2, 0.4)	< 0.0001		0.2 (0.1, 0.3)	< 0.0001		0.6 (0.4, 0.8)	< 0.0001		0.4 (0.2, 0.6)	< 0.0001		0.4 (0.2, 0.5)	< 0.0001	
Female	0.2 (0.1, 0.3)	< 0.0001		0.1 (0.1, 0.2)	< 0.0001		0.4 (0.3, 0.5)	< 0.0001		0.4 (0.3, 0.5)	< 0.0001		0.2 (0.1, 0.3)	< 0.0001	
Education al level			0.7			0.33			0.76			0.39			0.14
Illiterate	0.3 (0.2, 0.5)	< 0.0001		0.2 (0.2, 0.3)	< 0.0001		0.7 (0.5, 0.9)	< 0.0001		0.5 (0.3, 0.6)	< 0.0001		0.4 (0.2, 0.6)	< 0.0001	
Middle school or below	0.2 (0.2, 0.3)	< 0.0001		0.1 (0.1, 0.2)	< 0.0001		0.5 (0.3, 0.6)	< 0.0001		0.4 (0.2, 0.5)	< 0.0001		0.2 (0.1, 0.4)	< 0.0001	
High school or above	0.2 (0.1, 0.4)	0.01		0.2 (0.1, 0.3)	0.002		0.4 (0.1, 0.7)	0.01		0.5 (0.1, 0.8)	0.01		0.4 (0.1, 0.6)	0.003	
Marital status			0.13			0.17			0.12			0.08			0.38
Never married	0.5 (−1.0, 2.0)	0.48		0.3 (−0.6, 1.2)	0.52		1.2 (−1.4, 3.8)	0.35		3.5 (0.2, 6.7)	0.04		0.6 (−1.6, 2.8)	0.59	
Married	0.3 (0.2, 0.3)	< 0.0001		0.2 (0.1, 0.2)	< 0.0001		0.5 (0.4, 0.7)	< 0.0001		0.4 (0.3, 0.5)	< 0.0001		0.3 (0.2, 0.4)	< 0.0001	
Divorced	0.1 (−0.1, 0.3)	0.2		0.1 (−0.0, 0.2)	0.23		0.3 (0.0, 0.6)	0.03		0.4 (0.1, 0.7)	0.01		0.1 (−0.1, 0.4)	0.31	
Locality of residence			0.94			0.99			0.61			0.79			0.59
Urban	0.2 (0.1, 0.3)	< 0.0001		0.1 (0.1, 0.2)	< 0.0001		0.5 (0.4, 0.7)	< 0.0001		0.4 (0.2, 0.5)	< 0.0001		0.3 (0.1, 0.4)	< 0.001	
Rural	0.3 (0.2, 0.4)	< 0.0001		0.2 (0.1, 0.2)	< 0.0001		0.6 (0.4, 0.7)	< 0.0001		0.5 (0.3, 0.6)	< 0.0001		0.3 (0.2, 0.5)	< 0.0001	
Region of residence			< 0.0001			< 0.0001			< 0.0001			0.31			< 0.0001
South	0.5 (0.4, 0.6)	< 0.0001		0.3 (0.2, 0.4)	< 0.0001		0.9 (0.7, 1.1)	< 0.0001		0.5 (0.4, 0.7)	< 0.0001		0.9 (0.6, 1.2)	< 0.0001	
North	0.2 (0.1, 0.3)	< 0.0001		0.1 (0.1, 0.2)	< 0.0001		0.4 (0.3, 0.5)	< 0.0001		0.4 (0.2, 0.5)	< 0.0001		0.3 (0.2, 0.3)	< 0.0001	
Self-reported smoking status			0.01			0.01			< 0.001			< 0.001			0.11
No	0.2 (0.1, 0.3)	< 0.0001		0.1 (0.1, 0.2)	< 0.0001		0.4 (0.3, 0.5)	< 0.0001		0.3 (0.2, 0.4)	< 0.0001		0.3 (0.2, 0.4)	< 0.0001	
Yes	0.4 (0.2, 0.5)	< 0.0001		0.2 (0.1, 0.3)	< 0.0001		0.8 (0.6, 1.0)	< 0.0001		0.7 (0.5, 0.9)	< 0.0001		0.4 (0.2, 0.6)	< 0.0001	
Alcohol consumption			0.92			0.83			0.69			0.59			0.52
No	0.2 (0.2, 0.3)	< 0.0001		0.2 (0.1, 0.2)	< 0.0001		0.5 (0.4, 0.6)	< 0.0001		0.4 (0.3, 0.5)	< 0.0001		0.3 (0.2, 0.4)	< 0.0001	
> 1 m onth	0.3 (0.1, 0.4)	< 0.0001		0.2 (0.1, 0.2)	< 0.0001		0.5 (0.3, 0.7)	< 0.0001		0.3 (0.1, 0.5)	0.02		0.4 (0.2, 0.6)	< 0.001	
< 1 m onth	0.3 (0.1, 0.5)	< 0.001		0.2 (0.1, 0.3)	< 0.001		0.7 (0.4, 1.1)	< 0.0001		0.7 (0.3, 1.1)	< 0.001		0.3 (0.0, 0.6)	0.04	
Self-reported health status			0.91			0.9			0.97			0.84			0.88
Poor	0.3 (0.2, 0.3)	< 0.0001		0.2 (0.1, 0.2)	< 0.0001		0.5 (0.4, 0.6)	< 0.0001		0.4 (0.3, 0.5)	< 0.0001		0.3 (0.2, 0.4)	< 0.0001	
Good	0.3 (0.2, 0.4)	< 0.0001		0.2 (0.1, 0.2)	< 0.0001		0.5 (0.3, 0.7)	< 0.0001		0.4 (0.2, 0.6)	< 0.001		0.3 (0.1, 0.5)	< 0.001	
Hypertension			0.99			0.74			0.8			0.76			0.71
No	0.3 (0.2, 0.4)	< 0.0001		0.2 (0.1, 0.2)	< 0.0001		0.6 (0.4, 0.7)	< 0.0001		0.4(0.3, 0.6)	< 0.0001		0.4 (0.3, 0.5)	< 0.0001	
Yes	0.2 (0.1, 0.3)	< 0.0001		0.1 (0.1, 0.2)	< 0.0001		0.5 (0.3, 0.7)	< 0.0001		0.4 (0.2, 0.5)	< 0.0001		0.2 (0.1, 0.4)	0.003	
Physical_disability			0.79			0.91			0.83			0.56			0.41
No	0.3 (0.2, 0.3)	< 0.0001		0.2 (0.1, 0.2)	< 0.0001		0.5 (0.4, 0.6)	< 0.0001		0.4 (0.3, 0.5)	< 0.0001		0.3 (0.21 0.4)	< 0.0001	
Yes	0.2 (−0.1, 0.6)	0.17		0.1 (−0.1, 0.3)	0.32		0.4 (−0.2, 1.0)	0.2		0.6 (−0.1, 1.2)	0.07		0.4 (−0.1, 0.9)	0.14	

The model adjusted for all covariates. age, sex, educational level, marital status, locality of residence, region of residence, smoking status, alcohol consumption, self-reported health status, hypertension, self-reported physical disability.

#### Cubic spline regression analysis.

**Fig 3:**
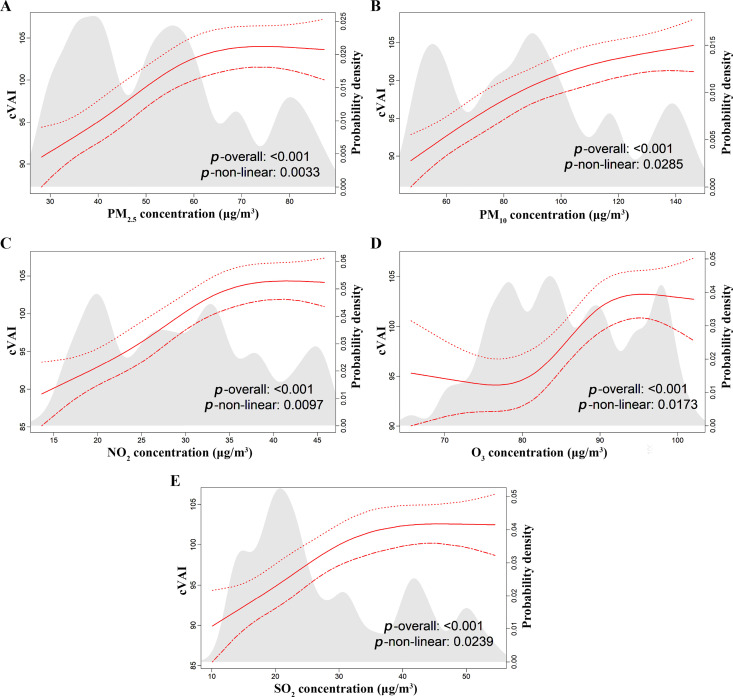
Curves showing a non-linear relationship between air pollutant concentrations and cVAI.

The restricted cubic spline analysis further identified a significant non-linear association between increasing levels of air pollutants (PM_2.5_, PM_10_, NO_2_, O_3_, and SO_2_) and the cVAI (*p* for non-linearity < 0.05, Fig 3). In particular, the non-linear relationship between O_3_ and cVAI was S-shaped, with the two inflection points being observed at 80 and 90 µg/m^3^, respectively (Fig 3).

### Contribution of individual pollutants to the total effect of outdoor air pollution on cVAI

**Fig 4:**
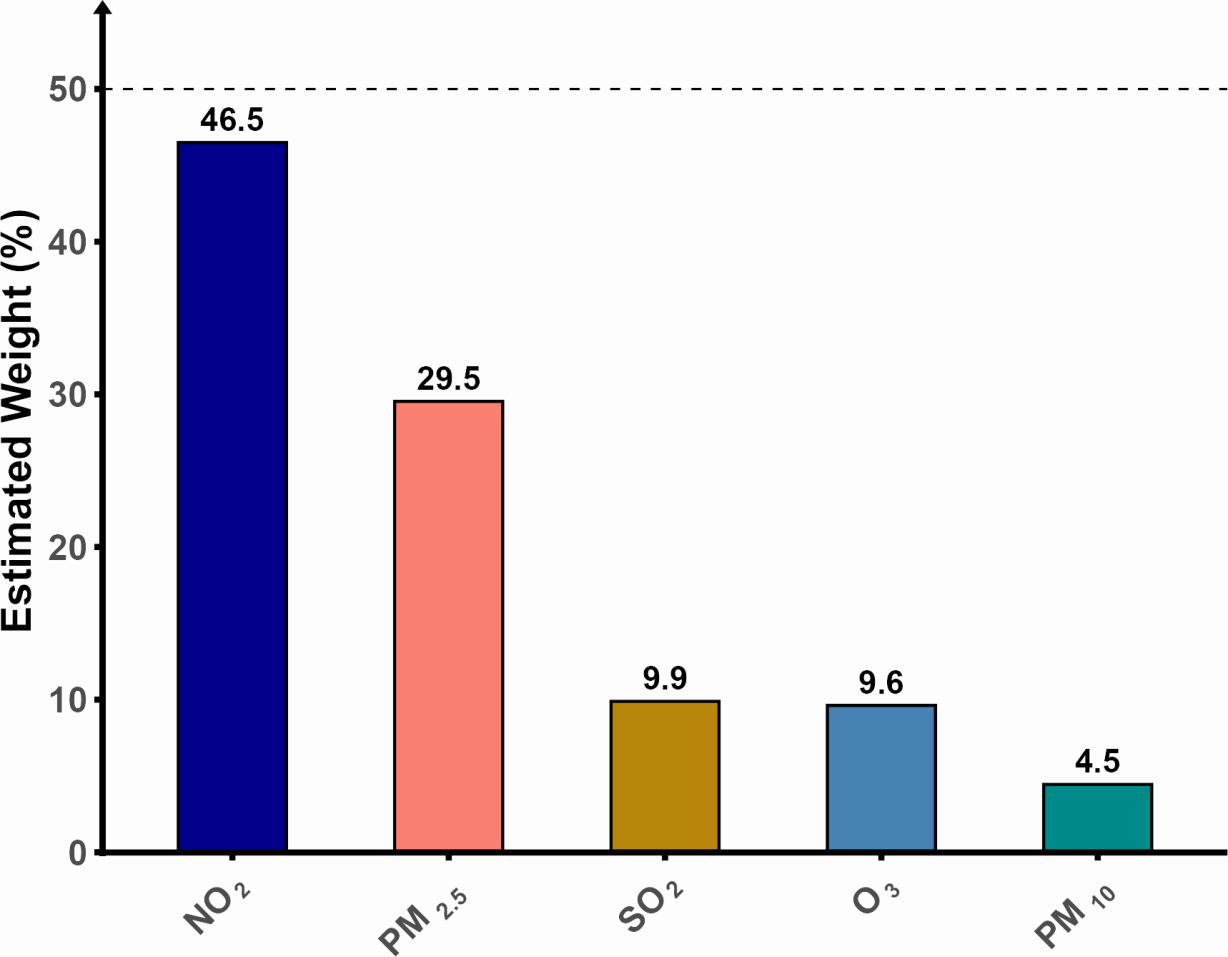
Results of the weighted quantile sum analysis of the contribution of individual pollutants to the combined effect of air pollution on cVAI.

The contribution of each pollutant to the total effect of outdoor air pollution on cVAI is indicated in Fig 4. NO_2_ and PM_2.5_ were the main contributors, driving 46.5% and 29.5% of the total effect of ambient air pollution, respectively.

## Discussion

This is, to the best of our knowledge, the first study to evaluate the association between the concentrations of multiple air pollutants (PM_2.5_, PM_10_, NO_2_, O_3_, and SO_2_) and cVAI in a nationally representative middle-aged and older adult population in China. Our results demonstrate a non-linear and a linear dose-response relationship between the ambient concentrations of all these pollutants and cVAI. In subgroup analyses, the magnitude of the associations appeared to be more pronounced in men than women for three pollutants (PM_2.5_, PM_10_ and NO_2_), in active smokers than in non-smokers for all pollutants except SO_2_, and in the South Region of China than in the North region for all pollutants (especially NO_2_), except O_3_. On WQS regression analyses, NO_2_ and PM_2.5_ appeared to drive 76.5% and 29.5% of the total effect of outdoor air pollution on cVAI.

A previous study [11], conducted in China, found that the ambient concentrations of PM_2.5_, PM_10_, CO, NO_2_, O_3_, and SO_2_ were significantly associated with increases in BMI and VAI values. This is consistent with our present findings linking the ambient concentrations of PM_2.5_, PM_10_, NO_2_, O_3_, and SO_2_ with cVAI. Other studies on the association of air ambient air pollutant concentrations with visceral fat in Asia further support our findings. For example, a Korean cross-sectional study found an association between PM_10_ and SO_2_ and a computerized tomography-based measure of abdominal fat [25,26]. Similarly, a UK Biobank study identified positive associations between outdoor air pollution exposure and the body fat percentage [27]. A Mendelian randomization study also found long-term exposure to ambient PM_2.5_ being associated with a 6.4-fold increased risk of obesity as well as with elevated visceral fat and elevated serum triglyceride levels in populations of European ancestry [28]. Furthermore, visceral adiposity was found to be associated with residency near major traffic roads in the Framingham Heart study, suggesting that traffic-related outdoor air pollution may alter the metabolic health perhaps by favoring the accumulation of fats in viscera [29].

The biological mechanisms linking outdoor air pollution exposure to visceral fat accumulation are complex, potentially involving inflammatory responses and oxidative stress among others [30]. The study of a mouse model of diet-induced obesity found that exposure to PM_2.5_ induces the infiltration of macrophages into visceral fat, thus triggering the release of inflammatory cytokines, such as interleukin-6 (IL-6) and tumor necrosis factor alpha (TNF-α), which accelerate the accumulation of visceral fat [31]. Chronic exposure of the mouse to PM_2.5_ also activates the unfolded protein response and adipogenesis-related genes, further worsening visceral fat accumulation and metabolic disorders [32]. Additionally, a study of C57BL/6 mice found that PM_2.5_ impairs mitochondrial functions in both visceral and brown adipose tissues, increasing oxidative stress and the risk of type 2 diabetes [33]. Another mouse study found that long-term exposure to inhaled O_3_ induces local lung and systemic oxidative stress and inflammatory responses, thus contributing to the accumulation of visceral fat and insulin resistance [34].

The higher likelihood of outdoor air pollutant concentrations to be associated with cVAI in men than in women and in active smokers than in non-smokers could be due to many factors including but not limited to the synergistic effects of smoking-induced oxidative stress and inflammation [35]. Our findings are dissident with data from a 2009 literature review which suggested that women could be more vulnerable to outdoor air pollution than men. However, that review focused primarily on respiratory diseases, lacked a direct analysis of objective individual exposure data, and relied mainly on data from more than 20 years ago which may no longer reflect the current social lifestyles or industrial advances [36]. On the other hand, a previous observational study found more unfavourable abdominal and visceral fat distribution in active smokers than in non-smokers [37]. A Mendelian Randomization study also identified a causal relationship between active smoking and visceral adiposity based on the waist-to-hip ratio [38]. Altogether, the higher frequency of active smoking in men than women (52.8% vs 6.2%) in this study suggests that the greater magnitude of the association between air pollutant concentrations and cVAI in men than women may be underpinned, at least in part, by active smoking. Indeed, cigarette smoking reportedly promotes the systemic release of reactive oxygen species and of pro-inflammatory cytokines such as IL-6 and TNF-α, thus putatively enhancing visceral fat accumulation [35].

In the present study, WQS analysis demonstrated that NO_2_ and PM_2.5_ were particularly involved in driving the accumulation of visceral fat in the context of exposure to multiple pollutants simultaneously, thus highlighting their potential key contributing role. These findings are consistent with those from previous studies linking outdoor air pollution with obesity and type 2 diabetes [39]. Among these two pollutants, studies have mainly linked PM₂.₅ exposure with premature mortality in older adults. However, the higher contribution of NO_2_ to the effect of air pollution in this study could partly be due to the stronger association between NO_2_ exposure and cVAI among residents of the South region than among those of the North region who were however exposed to higher levels of NO_2_ pollution than residents of the South region. This paradoxical finding suggests that the ambient temperature could have influenced the effects of NO_2_ on cVAI [40]. On the other hand, the finding of greater levels of air pollution in the North than in the South region of China may be explained by the presence of more heavy industrial cities and greater vehicle ownership in the North while southern cities are primarily characterized by the distribution of high-tech industries, and the pollutants from these industries mainly consist of nitrate and NO_2_ accumulating in the atmosphere [41–45].

Altogether, there is a need to curb the burden of visceral adiposity and related cardiometabolic disorders in middle-aged and older adults of Chinese ancestry through the reduction of ambient air pollution exposure. For researchers studying air pollution and cardiometabolic diseases, it is recommended to conduct predictive and explanatory cohort studies to assess whether there is a causal relationship between long-term outdoor air pollution exposure and cVAI. Further animal and experimental human studies should be conducted to explore the mechanisms linking air pollution exposure with cVAI and cardiometabolic diseases; and ultimately, research should aim to alleviate the impact of air pollution on cardiovascular health in middle-aged and older populations. For policymakers in China, it is recommended to establish policies and strengthen regulations to reduce the ambient concentrations of air pollutants (PM_10_, PM_2.5_, NO_2_, O_3_, SO_2_) to safe threshold levels, with particular emphasis on stricter regulation of NO_2_ emissions in the Southern region of the country. Some specific measures needed to reduce outdoor air pollution exposure include the expan sion of green spaces, the promotion of energy-efficient appliances, the encouragement of low-carbon transportation, and the provision of support to green industries. For healthcare providers, there may be a need to extend the dissemination of knowledge about the link between air pollution and both visceral fat and related diseases among the public to help them reduce their exposure to air pollution and therefore potentially prevent the onset or worsening of visceral obesity and associated cardiometabolic disorders. Individuals with visceral obesity may benefit from taking necessary measures to reduce their exposition to air pollution by implementing some measures in their daily lives such as the use of air purifiers and the increase of indoor greenery.

## Strengths and limitations

There are several strengths to our study. First, our findings are virtually generalizable to the national population of China given that they arose from the CHARLS cohort. However, due to the exclusion of some individuals because of missing data, our study sample may not fully represent the national middle-aged and older adult population. Second, unlike previous studies that predominantly focused on the relationship between individual air pollutants and obesity, diabetes or single cardiovascular diseases, we assessed the relationship between cVAI and combined and individual air pollutants because in real life, people are typically exposed to multiple air pollutants simultaneously. Furthermore, we adjusted for several possible confounding factors and conducted sensitivity analyses to ensure the robustness of results of the linear association between individual air pollutant concentrations and cVAI.

However, several limitations should also be considered. First, due to the protection of privacy information in the CHARLS database, we could only estimate participants’ outdoor air pollution exposure at the city level rather than at each participant’s residential address. This may have led to the underestimation of each participant’s level of air pollution exposure because of a lack of information about different sources of air pollution exposure including but not limited to traffic, working environments, and household activities. Accordingly, we had to narrow the scope of this study to the assessment of outdoor air pollution only. Future studies could consider assessing participants’ air pollution exposure using portable air pollution monitoring devices which are allegedly cost-effective holistic monitors of participants’ air pollution exposure in these kinds of epidemiological studies.

A second limitation of this study is the possibility that some confounding factors were left unchecked as is commonly observed when studying associations in observational studies. The potential residual confounding factors include not only the sources of air pollution not assessed, but also the dietary habits of study participants such as the consumption of food contaminated with pollutants and perhaps unexplored genetic predispositions to the association of ambient air pollution with cVAI. Third, the cross-sectional design of our study limits our ability to establish causality with regard to the observed associations. Fourth, chances are high that the self-reported covariates were affected by a recall bias. Along these lines, future studies should consider accessing the participants’ medical records to more reliably make the diagnosis of hypertension based on physicians’ diagnoses and objective patient information about antihypertensive drug treatment. Sixth, with the presence of various types of alcohol products with different concentrations on the market, determining only the amount of alcohol consumed may lead to biased results about alcohol consumption. Seventh, although our finding of peak weights for PM2.5 and NO2 on WQS analysis suggests that these pollutants played the most influencing roles in the association between air pollution and cVAI, air pollution is a complex mixture. The effects of different pollutants on the body may involve synergistic effects and potential collinearity, especially for the collinearity between PM_2.5_ and NO_2,_ which reportedly strong and influences findings about the respective contributions of each of the pollutants to the total effect of air pollution exposure. Notably, the effect of NO_2_ might have been overestimated.

## Conclusion

This study indicated that long-term outdoor exposure to the following air pollutants may be significantly associated with cVAI in middle-aged to older adults in China: PM_2.5_, PM_10_, NO_2_, O_3_ and SO_2_. NO_2_ and PM_2.5_ accounted for the majority of the effects of air pollution on cVAI and the harmful effects of outdoor air pollution exposure on cVAI were particularly pronounced in men and active smokers when compared with women and non-smokers, respectively. Future research should further investigate whether air pollutants and cVAI are causally linked. This study provided evidence for future environmental public health policies regarding outdoor air pollution exposure and visceral obesity.

## Supporting information

S1 Fig Flow chart showing how the study population was selected. (TIF)

S1 Table Sensitivity analyses: the quartiles of air pollutants’ concentrations and cVAI. (DOCX)

S1 File STROBE-checklist. (DOCX)
