# Supplementary figures and images for: The association between long-term outdoor air pollution exposure and Chinese visceral adiposity index: A nationwide study of middle-aged and older adults
Source: PLoS One. 2025 Jul 17;20(7):e0325524. doi: 10.1371/journal.pone.0325524 (PMC12270131; doi:10.1371/journal.pone.0325524)

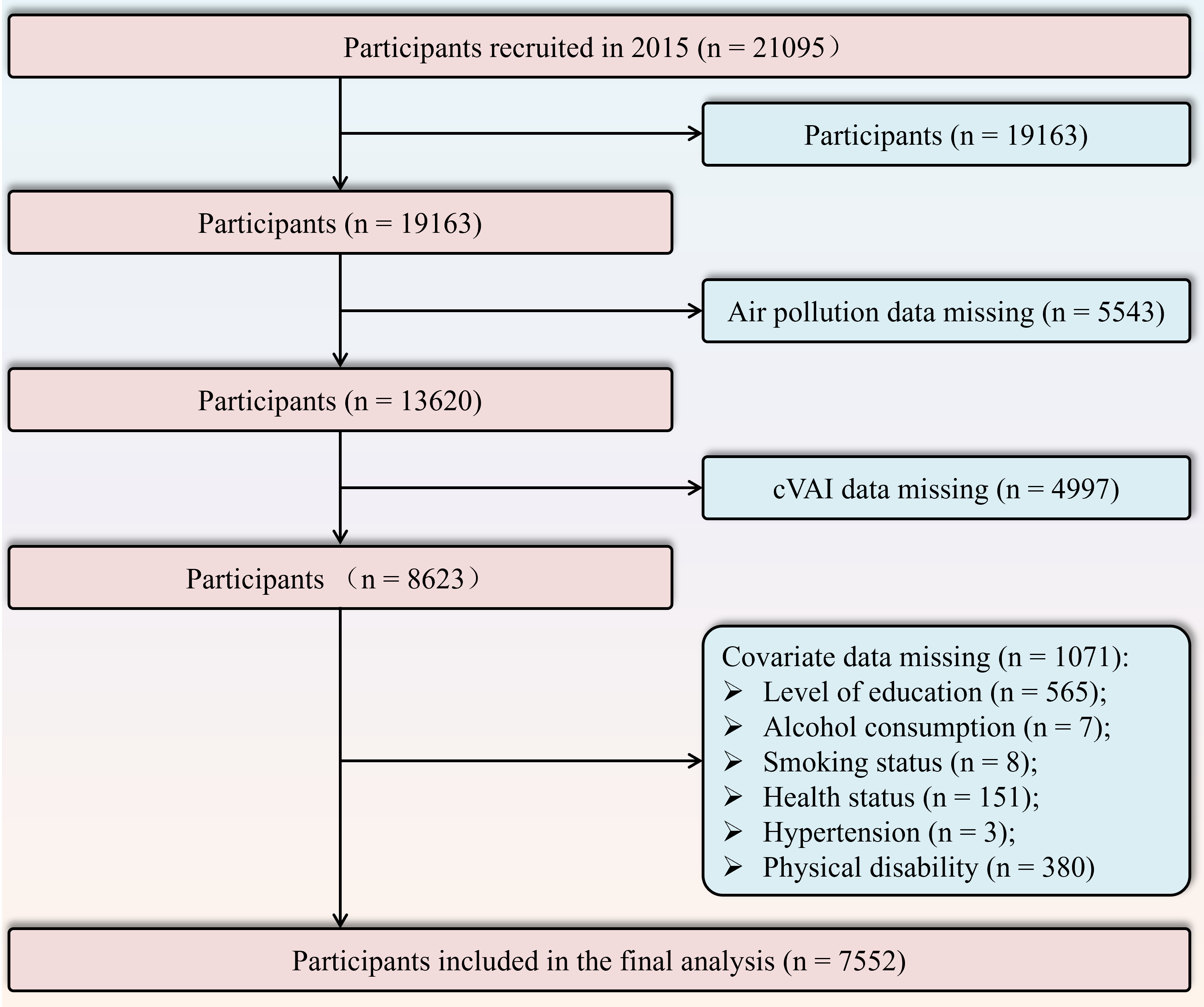

Supplement: S1 Fig — (TIF) [file pone.0325524.s001.tif]
