# Supplementary material for: The association between long-term outdoor air pollution exposure and Chinese visceral adiposity index: A nationwide study of middle-aged and older adults
Source: PLoS One. 2025 Jul 17;20(7):e0325524. doi: 10.1371/journal.pone.0325524 (PMC12270131; doi:10.1371/journal.pone.0325524)
Supplement: S1 Table — (DOCX) [file pone.0325524.s002.docx]

**Table S1.** Sensitivity analyses: the quartiles of air pollutants' concentrations and cVAI.

|  |  |  | | **cVAI** |  |  |
| --- | --- | --- | --- | --- | --- | --- |
|  | β (95%CI) | β (95%CI) | | β (95%CI) | β (95%CI) | β (95%CI) |
| Stage 1 sensitivity analysis | | | |  |  |  |
| Air polutants | **PM_2.5_** | | **PM_10_** | **NO_2_** | **O_3_** | **SO_2_** |
| Q1 | ref | | ref | ref | ref | ref |
| Q2 | 3.5 (1.9, 5.0) | | 0.6 (-0.9, 2.2) | 1.3 (-0.3, 2.8) | 1.4 (-0.1, 3.0) | 2.9 (1.4, 4.4) |
| Q3 | 5.1 (3.6, 6.6) | | 3.5 (1.9, 5.0) | 5.7 (4.2, 7.2) | 3.5 (1.8, 5.1) | 2.9 (1.3, 4.4) |
| Q4 | 3.8 (2.2, 5.5) | | 2.6 (0.9, 4.3) | 3.9 (2.2, 5.5) | 3.1 (1.5, 4.7) | 3.0 (1.2, 4.8) |
| p for the trend | < 0.0001 | | < 0.0001 | < 0.0001 | < 0.0001 | 0.001 |
| Stage 2 sensitivity analysis | | | |  |  |  |
| Air polutants | 95% CI | | 95% CI | 95% CI | 95% CI | 95% CI |
| Q1 | ref | | ref | ref | ref | ref |
| Q2 | 5.6 (3.3, 8.0) | | 3.1 (0.8, 5.4) | 3.8 (1.5, 6.1) | -0.9 (-3.3, 1.5) | 5.3 (3.0, 7.6) |
| Q3 | 9.4 (7.1, 11.7) | | 8.4 (6.1, 10.7) | 11.9 (9.5, 14.2) | 6.0 (3.5, 8.5) | 7.1 (4.7, 9.5) |
| Q4 | 12.1 (9.5, 14.6) | | 11.3 (8.7, 13.9) | 12.0 (9.5, 14.4) | 6.7 (4.2, 9.1) | 10.9 (8.1, 13.7) |
| p for the trend | < 0.0001 | | < 0.0001 | < 0.0001 | < 0.0001 | < 0.0001 |

Abbreviation: CI: confidence interval; Ref: reference.
